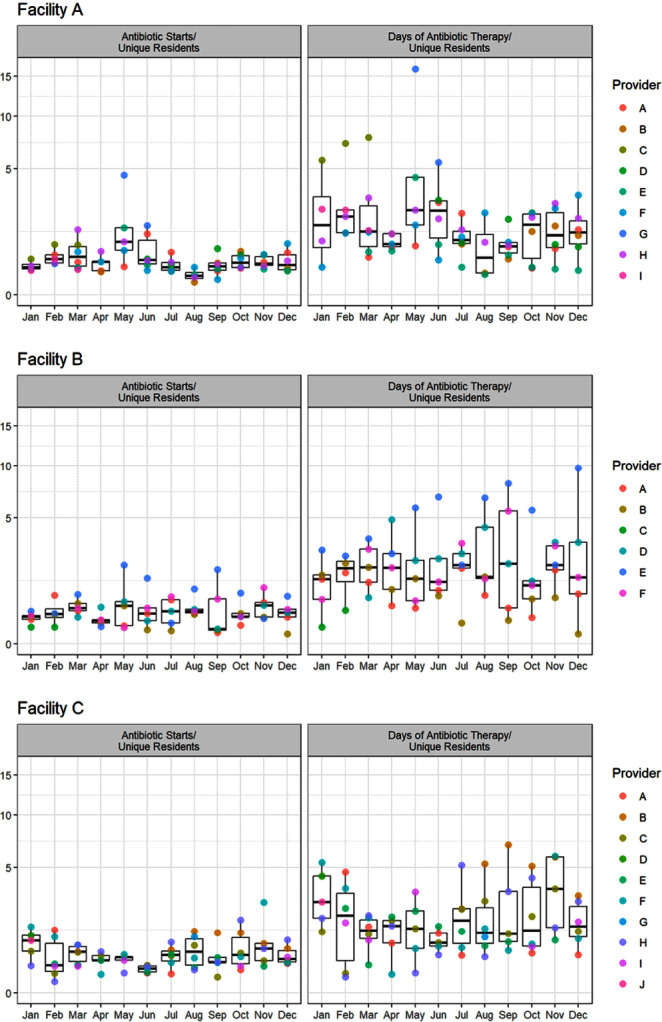# Two Novel Antibiotic Use Metrics for Facilities and Individual Prescribers in Post-Acute and Long-Term Care Settings

**DOI:** 10.1017/ash.2024.131

**Published:** 2024-09-16

**Authors:** Sunah Song, Brigid Wilson, Taissa Bej, Corinne Kowal, Federico Perez, David Nace, Robin Jump

**Affiliations:** Institute for Computational Biology; Northeast Ohio VA Healthcare System; Department of Veteran Affairs; University of Pittsburgh; VA Pittsburgh Healthcare System

## Abstract

**Background:** Measuring and reporting antibiotic use are essential to antimicrobial stewardship activities. The most common metric to assess facility-level use is days of antibiotic therapy per 1000 days of care (DOT/1000 DOC). This metric may be difficult to calculate, not be readily comparable, or not provide actionable data to individual prescribers, particularly those that work in post-acute and long-term care (PALTC) settings. Here we use data from a centralized dispensing pharmacy to develop antibiotic use metrics suitable for offering individualized feedback to prescribers working in PALTC settings. **Methods:** We obtained medication dispensing data and resident census data for 13 PALTC settings within the same network. After omitting non-pharmacologic items and limiting the data to medications dispensed from 1/2020 – 12/2022, we determined the following metrics by month: days of antibiotic therapy (DOT), number of medications prescribed, number of antibiotic courses prescribed (antibiotic starts), and the number of individual residents issued a prescription for any medication (unique residents). These metrics were assessed for each facility (2020 – 2022) and for prescribers responsible for > 1% of prescriptions within that facility (2022 only). Prescriber-level unique residents was the number of residents issued a prescription by the given provider. We obtained facility-level census data to calculate antibiotic DOT/1000 resident days of care (DOC) as a standard to which we compared novel metrics. **Results:** During the 3-year study period, 1718 prescribers at 13 PALTC settings wrote for 672256 medications, including 31087 antibiotic courses. At the facility level, the correlation between monthly antibiotic starts (courses)/unique residents and antibiotic DOT/1000 DOC was 0.83 (p < 0 .0001). The correlation between monthly antibiotic DOT/unique residents and antibiotic DOT/1000 DOC was 0.98 (p < 0 .0001). Trends in monthly values of both novel metrics and DOT/1000 DOC were consistent across the examined period (Figure 1). For individual prescribers, both novel metrics permit assessment and comparison of antibiotic prescription rates over time (Figure 2). **Conclusions:** Pharmacy dispensing data can be used to determine antibiotic DOT/unique residents and antibiotic starts/unique residents at the facility level and for individual providers. The novel metric antibiotic DOT/unique residents demonstrated strong correlation with antibiotic DOT/1000 DOC at the facility level. In addition to supporting tracking and reporting of antibiotic use among PALTC settings, these new metrics permit visualization of the antibiotic prescribing rates of individuals prescribers, as well as peer comparison, which in turn can lead to actionable feedback that helps improve antibiotic use in the care of PALTC.

**Figure:**
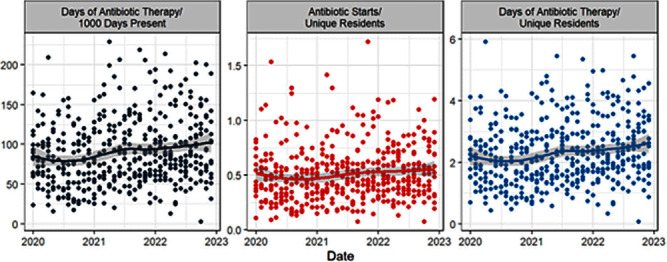

**Figure:**